# Antigen Uptake during Different Life Stages of Zebrafish (*Danio rerio*) Using a GFP-Tagged *Yersinia ruckeri*

**DOI:** 10.1371/journal.pone.0158968

**Published:** 2016-07-12

**Authors:** Rozalia Korbut, Foojan Mehrdana, Per Walter Kania, Marianne Halberg Larsen, Dorte Frees, Inger Dalsgaard, Louise von Gersdorff Jørgensen

**Affiliations:** 1 Laboratory of Aquatic Pathobiology, Department of Veterinary Disease Biology, University of Copenhagen, Frederiksberg, Denmark; 2 Food Safety and Zoonoses, Department of Veterinary Disease Biology, University of Copenhagen, Frederiksberg, Denmark; 3 Section for Bacteriology and Pathology, National Veterinary Institute, Technical University of Denmark, Frederiksberg, Denmark; INRA, FRANCE

## Abstract

Immersion-vaccines (bacterins) are routinely used for aquacultured rainbow trout to protect against *Yersinia ruckeri* (Yr). During immersion vaccination, rainbow trout take up and process the antigens, which induce protection. The zebrafish was used as a model organism to study uptake mechanisms and subsequent antigen transport in fish. A genetically modified Yr was developed to constitutively express green fluorescent protein (GFP) and was used for bacterin production. Larval, juvenile and adult transparent zebrafish (tra:nac mutant) received a bath in the bacterin for up to 30 minutes. Samples were taken after 1 min, 15 min, 30 min, 2 h, 12 h and 24 h. At each sampling point fish were used for live imaging of the uptake using a fluorescence stereomicroscope and for immunohistochemistry (IHC). In adult fish, the bacterin could be traced within 30 min in scale pockets, skin, oesophagus, intestine and fins. Within two hours post bath (pb) Yr-antigens were visible in the spleen and at 24 h in liver and kidney. Bacteria were associated with the gills, but uptake at this location was limited. Antigens were rarely detected in the blood and never in the nares. In juvenile fish uptake of the bacterin was seen in the intestine 30 min pb and in the nares 2 hpb but never in scale pockets. Antigens were detected in the spleen 12 hpb. Zebrafish larvae exhibited major Yr uptake only in the mid-intestine enterocytes 24 hpb. The different life stages of zebrafish varied with regard to uptake locations, however the gut was consistently a major uptake site. Zebrafish and rainbow trout tend to have similar uptake mechanisms following immersion or bath vaccination, which points towards zebrafish as a suitable model organism for this aquacultured species.

## Introduction

Aquacultured fish are vaccinated at different life stages in order to confer protection against various diseases. The administration of vaccines depends on the size of the fish. Injection is problematic in small fish, but immersion vaccines can be administered when fish weigh from 0.5 to 10 g, where the adaptive immune system has become responsive [1]. Immersion vaccines (bacterins) are gentle and thus ideal for small fish. For rainbow trout (*Oncorhynchus mykiss*), commercial immersion vaccines are available against enteric redmouth disease caused by the bacterium *Yersinia ruckeri* (Yr) [2,3]. The initial route of entry, following immersion into a Yr bacterin of rainbow trout, has been investigated [4,5] and it was found that antigens were taken up primarily by the skin, gills and gut [4,5], from where bacterial antigens were actively transported to immune organs. The major part of antigen uptake was, however, found to take place at different sites during different life stages of rainbow trout. Fry (0.32 g) were found to take up Yr antigens primarily via the gills and gut, whereas larger fish (4–5 g) were found primarily to take up the antigens via the gut and skin.

In this study, the zebrafish (*Danio rerio*) was used as a model organism for rainbow trout to study the uptake of a bacterin. Zebrafish is a member of the family Cyprinidae and rainbow trout of the Salmonidae and these families separated evolutionary 110–160 million years ago [6,7]. This separation, perceived from an evolutionary point of view, took place not long ago. The fish are, however, different in several aspects. Rainbow trout are carnivores and thrive at 15°C, whereas zebrafish are omnivores and prefer higher temperatures (∼28°C). Common for both species is the fact that they are teleosts and live in freshwater, which present an argument that zebrafish may be well suited as a powerful model organism for rainbow trout.

The zebrafish is already a well-established model organism for human diseases [8]. It has been used as a model organism for research areas such as biomedicine [9,10], cancer research [11], toxicology studies [12], developmental biology [13] and genetics [9]. Furthermore, it has served as a model organism to get a better understanding of the vertebrate immune system [14–16].

Zebrafish has several advantages compared to other fish species. It is easy to handle and its complete genome is sequenced and well mapped. It is characterised as a small size fish: adult zebrafish may reach a body length of 38 mm and a weight up to 0.9 g. It has a short generation time, reaches maturity within 90 days post fertilization (dpf), it generates large number of offspring and has external fertilization and embryonic development [7]. Many mutants and transgenic strains with different traits and characteristics are available. Whole body analyses of all life stages of zebrafish are therefore easily accessible.

Recently, zebrafish have become more popular as a model organism for aquaculture research and has been implemented as a good infection model for aquaculture-associated pathogens [7,17,18]. Studies in zebrafish concerning infections with *Aeromonas hydrophila* and *A*. *salmonicida* [19–21], vaccine and infection studies against viral hemorrhagic septicemia virus (VHSV) [22] and bath vaccinations with live attenuated *Vibrio anguillarum* [23] are all of relevance for rainbow trout. Zebrafish has also been used in aquaculture research within nutrition [24–26], probiotics [27] and stress [7].

Here, we investigated the antigen uptake in double mutated transparent zebrafish (tra:nac [28,29]), following a bath vaccination into a bacterin consisting of inactivated GFP-tagged Yr. Uptake sites and mechanisms were evaluated during different life stages of zebrafish, in order to evaluate differential uptake mechanisms and correlate these to studies conducted in rainbow trout [4,5]. Furthermore, to avoid bias from tissue manipulations, a method was developed, where uptake sites in live fish could be visualized.

## Materials and Methods

### Ethics statement

All experiments have been conducted according to a permit obtained from The Animal Experiments Inspectorate under the Danish Ministry of Environment and Food (Permit: 2014-15-0201-000-25). During anaesthesia and euthanization tricaine methanesulphonate (MS222) was used. No animals became ill or died prior to the experimental endpoint.

### Genetically modified *Y*. *ruckeri*

Yr (serotype O1, biotype 2) was transfected with the plasmid pNF8, containing the erythromycin (ERY) resistance gene and the GFP sequence [30] (= YrpNF8), using electroporation. Briefly described; a 250 mL batch of Yr in LB medium was grown at room temperature (RT) until exponential growth at OD 0.5–0.7. The cells were spun down and washed 3 times in 50 mL ice-cold 10% glycerol. After the last spin the cells were eluted in 250 μl ice-cold 10% glycerol and aliquoted into sterile eppendorf tubes with 50 μL in each and stored at -80°C until use. 1, 2 and 3 μl of the purified plasmid were added to a vial of Yr cells, respectively and carefully mixed on ice. The vials were left for 30 min on ice and subsequently transferred to precooled cuvettes (2 mm). Electroporation was conducted at 25 μF, 2.3 kV and 200 mΩ. Immediately, 900 μL pre-warmed LB medium was added and cells were transferred to eppendorf tubes and left for two hours at RT in a Queue^**®**^ Orbital shaker at 180 rpm. Cells were spread out on an agar plate containing ERY and grown for two days at RT. Colonies were confirmed for the correct insert by PCR and the ability to emit green light using a fluorescence microscope (Olympus, model BH2-RFL) using BP-490 nm illumination.

### Bacterin production

Overnight cultures of YrpNF8 were used to induce new cultures grown in 250 mL of LB medium including ERY for two days on the Queue^**®**^ Orbital shaker (180 rpm) at RT. Cells were spun down and washed twice in 100 mL ice-cold PBS (pH 7.4). Then the cells were formalin inactivated for 4 h in neutral formalin buffer 10% (Hounisen, Denmark) at RT with slow stirring. Following inactivation cells were washed twice in 100 mL ice-cold PBS and dissolved in 250 mL of water from the zebrafish facility in order to keep the water conditions constant for the fish. Two hundred μL were taken out in triplicate and spread out on selective agar plates, which were incubated for 48 hours at 20°C to check for no survivors of YrpNF8.

### Fish

Transparent zebrafish (tra:nac) [31,32], with a strong reduction of iridophores (transparent (tra)) [29] and a lack of melanophores (nacre (nac)) [28] were obtained from the Max Planck Institute for Developmental Biology, Tübingen, Germany. They were reared in a recirculated system (Aquaschwarz, Germany) at 28°C with a pH of 7.4 and conductivity of 550 μS. Ten percent of the water was changed every day and the fish were fed with live Artemia and pelleted dry feed (ZM Fish Food, England) one to three times per day. Larvae (20 dpf), juveniles (54 dpf) and adult fish (90 dpf) were used for this study.

### Bath and sampling

Three different age groups of 30 fish each were bathed in a bacterin with a maximum exposure of 30 min (two samplings were conducted before the full exposure) and then transferred to clean zebrafish facility water. Adult zebrafish experiments were run in duplicate and the larval experiments were repeated. Five control fish from each group were sham bathed for 30 min into 250 mL pure zebrafish facility water and then transferred to another container with clean zebrafish facility water. Sampling was conducted at 1 min, 15 min, 30 min, 2 h, 12 h and 24 hpb. At each sampling point five fish were sampled per group, two for live imaging and three for IHC.

### Live imaging

Fish were anaesthetised in tricaine methanesulphonate (MS222) (Sigma, Denmark) at a concentration of 100 mg/L and studied using a dissection microscope with GFP filter settings (Leica MZ FLII). An external visual investigation was conducted and images were taken of all parts of the fish at different magnifications. Subsequently, the fish was euthanized in a concentration of 300 mg/L MS222. Autopsy was conducted by removing the left belly flap exposing organs. Liver, intestine and spleen were taken out, carefully separated and photographed using the GFP filter settings. Finally, the gills and head kidney were removed and images taken.

### IHC

Fish were euthanized in a concentration of 300 mg/L MS222. The tail region posterior to the anus was removed with a scalpel, the peritoneal cavity cut open ventrally and parts fixed in formalin at RT for 24 h with slow stirring. Thereafter, the fish were transferred to 70% ethanol until dehydration and embedding in paraffin. Paraffin embedding followed a modified protocol [33] with serial baths of 50 min in each of 70% ethanol, 2 x 96% ethanol, 2 x 99% ethanol, 2 x xylene and 2 x paraffin to dehydrate the sample and prepare them for embedding.

Blocks were sectioned on a microtome (Leica RM2135) with sections of 4 μm. The sections were placed on a slide and dried at 40°C overnight (ON). Thereafter, the tissue was rehydrated in a series of baths; 2 x 5 min in xylene, 2 x 3 min in 99% ethanol, 2 x 3 min in 96% ethanol, 2 min in tris-buffered saline (TBS, pH 7,5). The tissue was blocked in 1.5% H_2_O_2_ in TBS for 10 min and subsequently washed in tab water for 5 min.

#### Antigen retrieval

The tissue was placed at 85°C for two hours in sodium citrate buffer (2.94 g Tri-Sodium Citrate + 0.5 mL Tween 20 + distilled water added to 1 L, pH 6.0) and subsequently left for 15 min to cool down. The sodium citrate was washed out with tab water for 5 min.

#### Staining

Sections were blocked in 2% bovine serum albumin (BSA) in TBS for 10 min. The blocking buffer was gently tabbed off and the tissue was incubated with a monoclonal anti-*Y*. *ruckeri* antibody (IBT, Lot#TO129, Germany) in a concentration of 1:5000 and left overnight (ON) at 4°C. The primary antibody was gently tabbed off and the slides washed in 3 changes of TBS. The tissue was incubated with an amplifier (Cell Marque, HiDefDetection^TM^ HRP Detection System, AH-Diagnostics, Denmark) for 10 min and slides were washed in 3 changes of TBS. Thereafter, the tissue was incubated with secondary antibodies for 10 min and washed in 3 changes of TBS. Finally, the slides were placed for 15 min in a carbazole bath (200 mL acetic acid buffer + 100 μL 30% H_2_O_2_ + 10 mL stock solution of carbazole (0.800 g 3-amino-9-ethyl carbazole + 100 mL acetone)) to acquire the colour reaction. Tissues were counter stained with Mayer's haematoxylin (Dako, Denmark) for 35 sec and mounted with aquatex (Merck, Denmark). Slides were examined using an automated DM5000 B microscope (Leica, Denmark). The whole fish was examined for colour reactions and pictures were taken of relevant tissues using LAS v4.3 software.

Alternatively, following the primary antibody stain, slides were washed 2 x 5 min in TBS and incubated in a dark chamber with a secondary anti-mouse antibody conjugated to a fluorophore, Dylight^TM^ 488 (ThermoScientific, Denmark) at RT for 30 min in a concentration of 1:250 in PBS. The slides were subsequently washed 2 x 5 min in TBS and mounted with Vectashield containing DAPI (Vector Laboratories, USA). Slides were examined using an SP5-X confocal microscope using GFP filter settings (Leica, Denmark).

## Results

In order to detect antigen uptake in zebrafish, a transparent strain was submersed in a formalin-inactivated soup of GFP-tagged *Y*. *ruckeri* and for detection of antigen uptake two different strategies (IHC and live imaging) were applied.

### Bacterin uptake in zebrafish tissues using IHC

#### Adults

**Fig 1:**
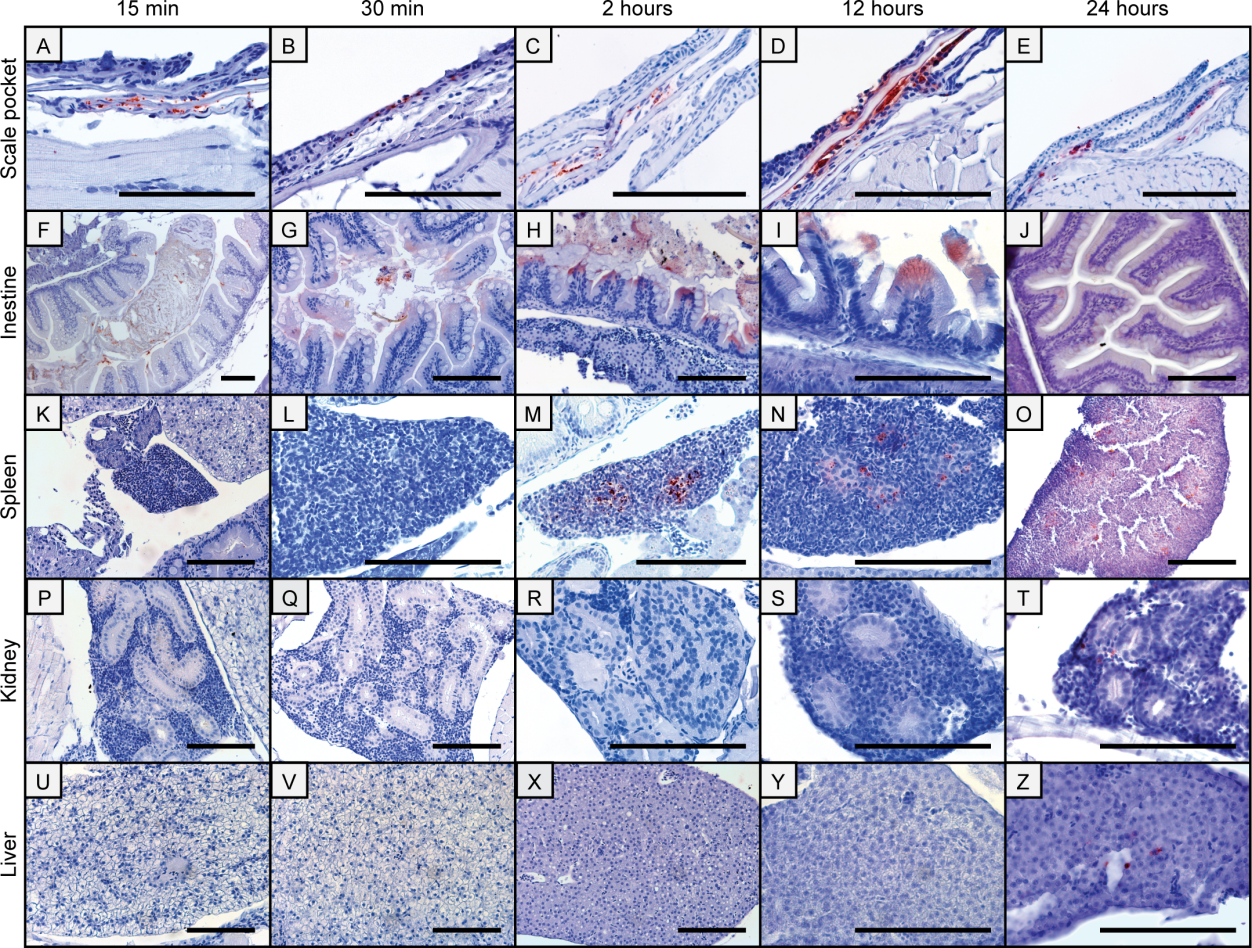
Immunohistochemical images of antigen uptake in adult zebrafish tissues following bath in a *Y*. *ruckeri* bacterin. Antigen staining is visible in scale pockets (A-E) and in the intestines from 15 min to 24 hpb (F-J). Two to 24 hpb the Yr bacterin is present in the spleen (M-O) and 24 hpb staining of the bacterin can be observed in the kidney (T) and in the liver (Z). All scale bars are 100 μm long.

Uptake was immediately detected in skin and/or scale pockets of the adult transparent zebrafish. It was detected one minute post bath (pb) in the epidermis and in the dermal layer in scale pockets. Throughout all sample points bacterin was detectable in the scale pockets (Fig 1A–1E (one minute not shown)). From 30 min and onwards no bacterin was found on the surface of the skin but occasionally in the cells in the epidermal layer. From 30 min to 24 hours, cells in the skin stained positive for the bacterin, and it was visible in deeper dermal layers (Fig 1B–1E). The bacterin was never seen in the muscle layer.

Uptake of the bacterin was also observed in the intestine of the adult fish from 1 min (not shown) pb persisting until the end of the study (Fig 1F–1J). At 1 and 15 min the bacterin was mainly detected in the intestinal bulb (anterior intestine) (Fig 1F). At later time points the bacterin was visible at the posterior part of the intestine including the anus. The bacterial antigen was visible inside enterocytes from 15 min following bath until the end of the study. Enterocytes of the anterior intestine (intestinal bulb) did not stain positive for the bacterin, however, enterocytes in the mid intestine and posterior intestine did. Uptake of the bacterin was visually most pronounced at 30 min, 2 hours and 12 hours (Fig 1G–1I).

**Fig 2:**
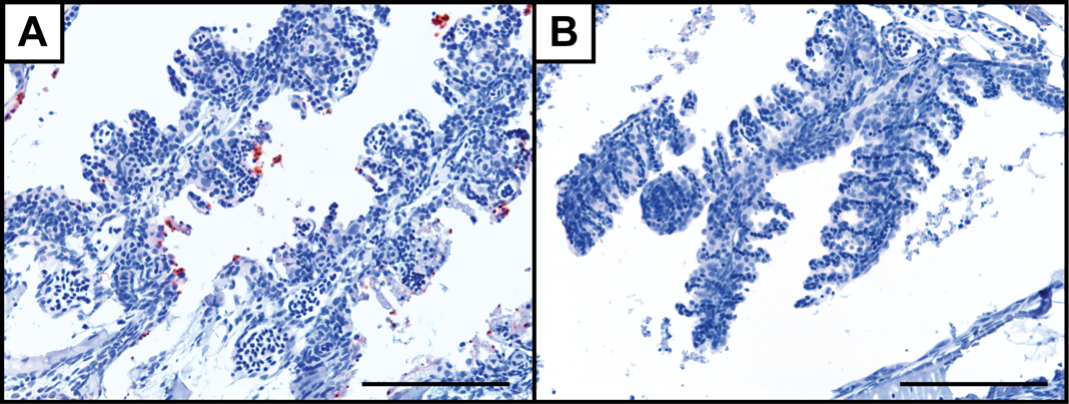
Immunohistochemical visualisation of *Y*. *ruckeri* bacterin in the gills of adult zebrafish following bath. Staining of a *Y*. *ruckeri* bacterin is observed in the gills of an adult zebrafish 15 min (A) and 30 min (B) following bath. The scale bars are 100 μm long.

From two hours following bath to the end of the study the bacterin was visible in the spleen (Fig 1M–1O) and it appeared in the kidney and the liver 24 hpb (Fig 1T and 1Z). At all sampling points the bacterin was detected on gill filament surfaces (Fig 2A) but with low intensity or nearly absence at 30 min pb and onwards (Fig 2B). Occasionally, cells positive for the bacterin was seen at gill surfaces, but uptake on a larger scale was never recorded.

An overview of the locations of the relevant organs and tissues in the zebrafish is illustrated in S1 Fig. For staining of control tissues, please refer to S2 Fig.

#### Juveniles

**Fig 3:**
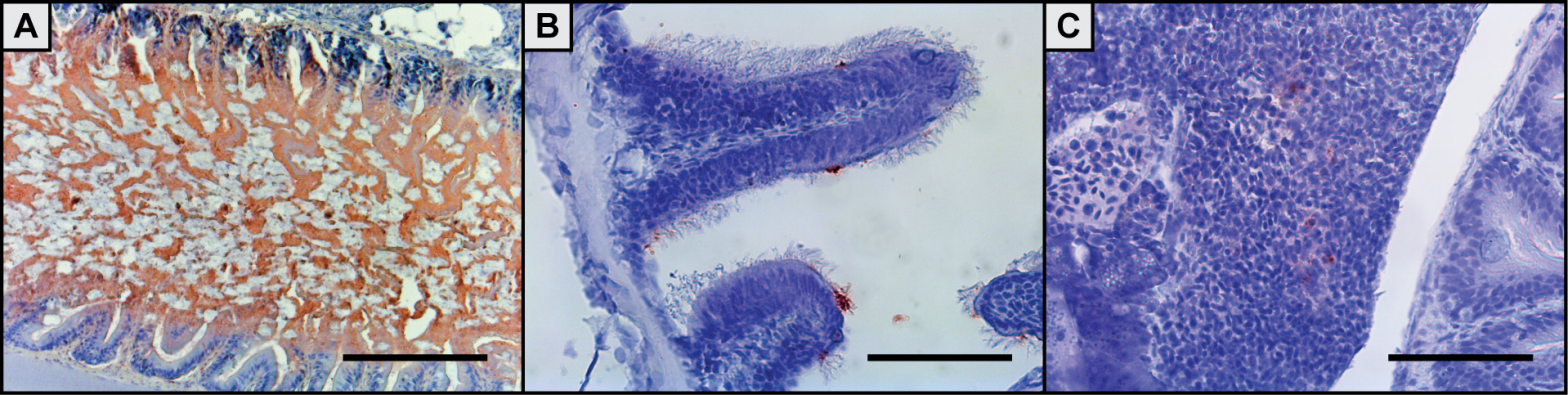
Immunohistochemical images of antigen uptake in juvenile zebrafish tissues following bath in a *Y*. *ruckeri* bacterin. Extensive bacterin uptake is seen in the intestine of juvenile zebrafish 30 min post bath (pb) (A). At the basis of the sensory hairs in the nares staining is observed two hpb (B) and the bacterin is subsequently present in the spleen 12 hpb (C). All scale bars are 100 μm long.

No or negligible staining was observed on the skin and it was never found in scale pockets. From 1 min pb (not shown) the bacterin was visible in the anterior part of the intestine and it was present in the intestine throughout the sampling period. At 30 min pb the uptake in the intestine was most pronounced and uptake was seen in enterocytes (Fig 3A). There was no presence of the bacterin in kidney, liver or gills at any time points, however it was observed at the basis of the sensory hairs in the nares at 2 hpb (Fig 3B) and in the spleen at 12 hpb (Fig 3C)

#### Larvae

**Fig 4:**
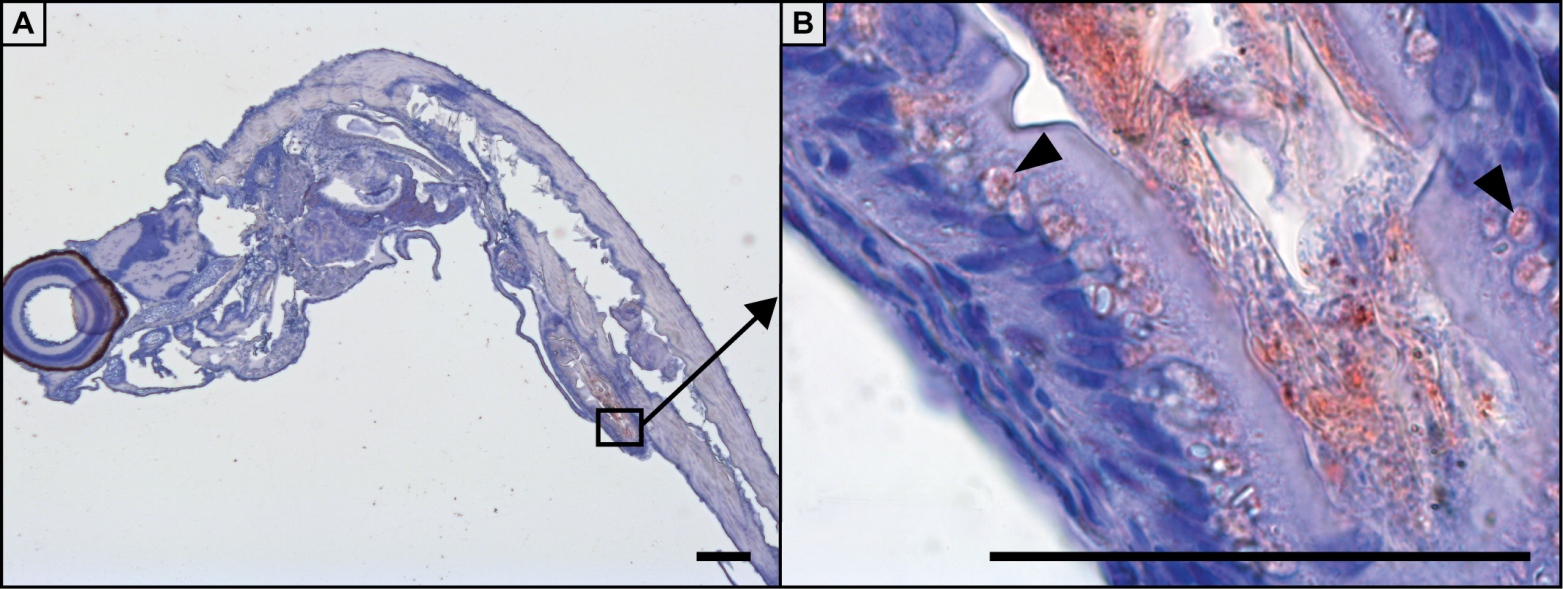
Immunohistochemical staining of antigen uptake in 20 dpf zebrafish larvae 24 h post bath with a *Y*. *ruckeri* bacterin. A whole larvae is shown (A), where specialised enterocytes of the mid-intestine has taken up the bacterin (B). The scale bars are 100 μm long.

**Fig 5:**
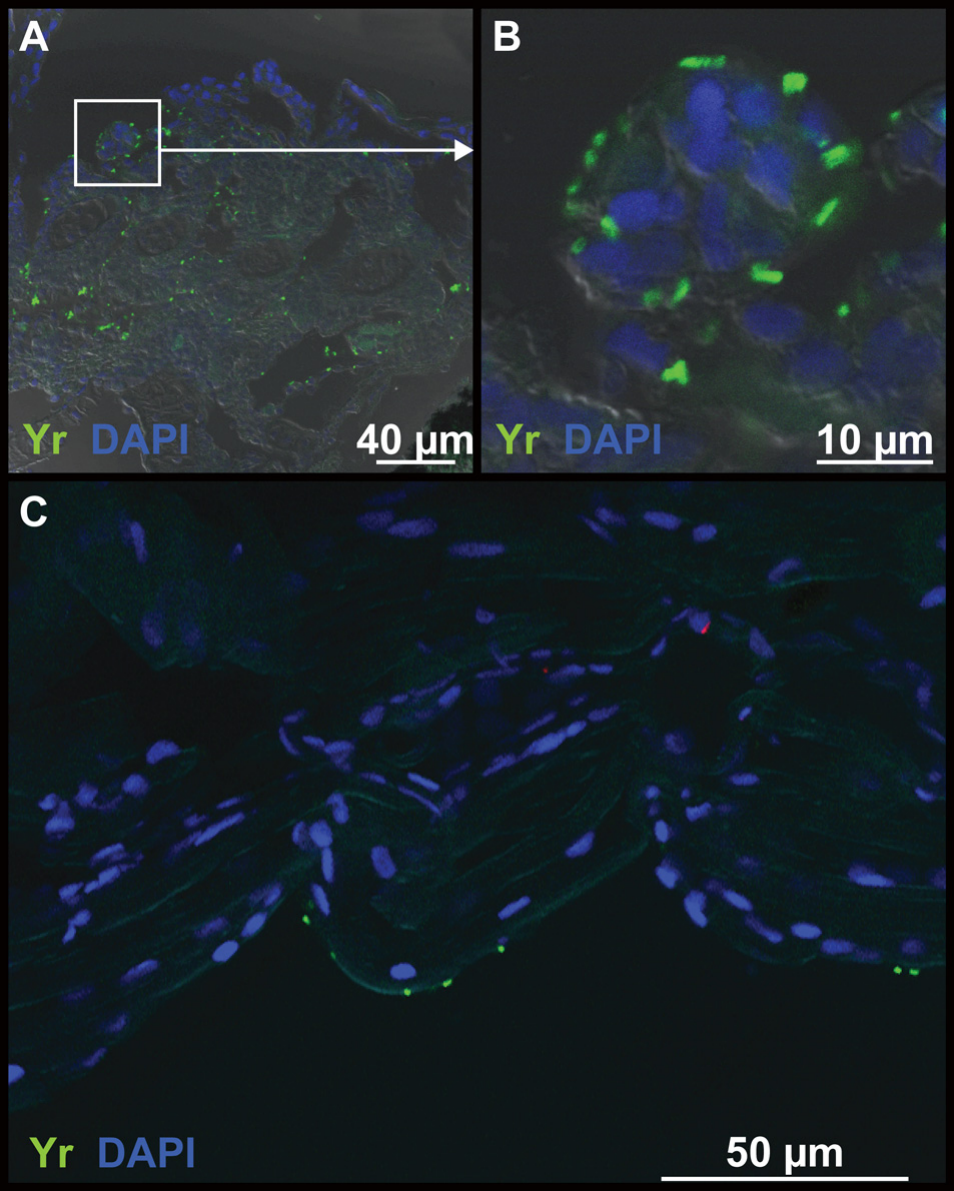
Immunohistochemical staining of the gills and skin of 20 dpf zebrafish larvae following bath with a *Y*. *ruckeri* bacterin. Fifteen min post bath the bacterin is present in the gills of zebrafish larvae (A and B) and also on the skin of the fish (C). Bacterin staining is green and nuclei staining is blue (DAPI).

Throughout the entire sampling period, uptake of the bacterin was only observed in the intestine. From 1 min until 12 h the intestinal bulb stained positive for the bacterin, however no uptake in cells was observed (not shown). At 24 hpb intestinal absorptive enterocytes in the mid-intestine stained positive for the bacterin (Fig 4A and 4B). When staining was conducted with fluorophores and visualized using a confocal microscope the bacterin was also found surrounding the gills (Fig 5A and 5B) and on the skin (Fig 5C) 15 min pb.

### Live imaging of GFP-tagged bacterin uptake in transparent zebrafish

#### Adults

**Fig 6:**
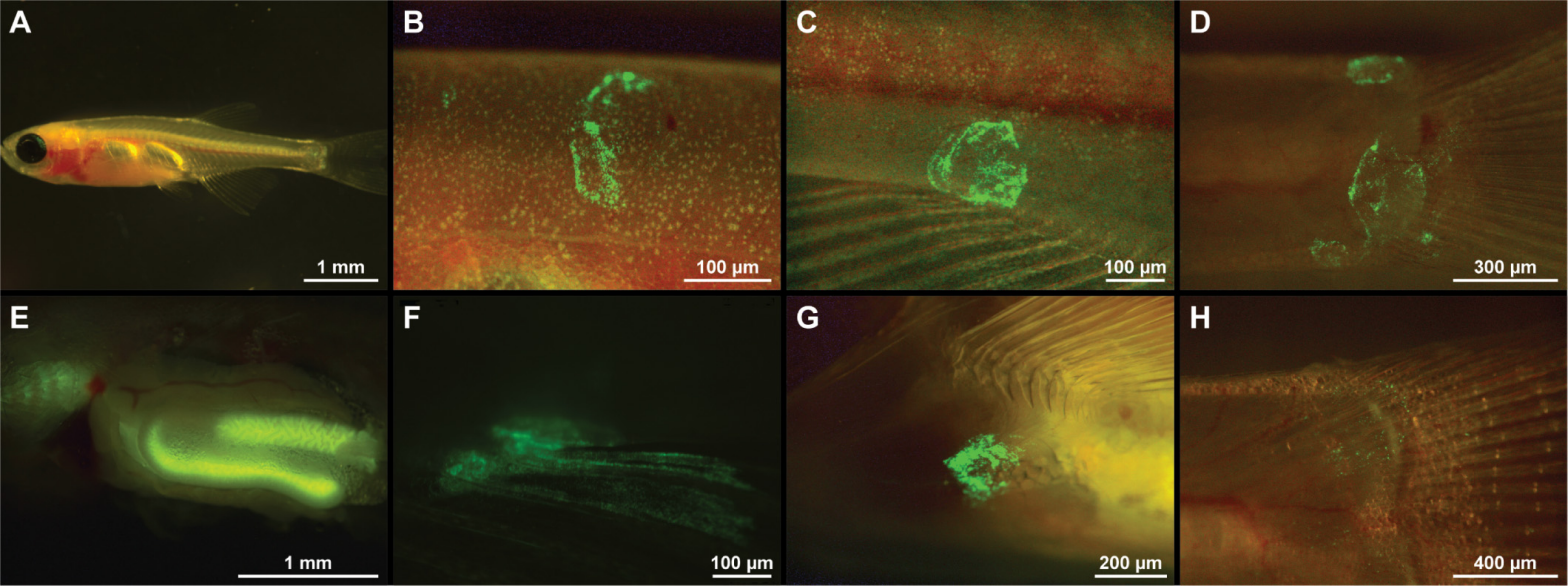
Images of antigen uptake in a transparent zebrafish following bath in a GFP-tagged *Y*. *ruckeri* bacterin. (A) The transparent tra:nac mutant, which was used for the investigations. (B) GFP-tagged *Y*. *ruckeri* bacterin on the skin of adults at one min post bath (pb). Bacterin was detected in scale pockets following 15 min (C) and 30 min pb (D). The GFP-tagged bacterin was observed 30 min pb in the intestine and in the oesophagus, which can be realised behind the gills (E). Presence of the bacterin was furthermore detected on the skin and in scale pockets following 30 min (F), 2 h (G) and 12 hpb (H).

Live imaging techniques applied on the outer surfaces of the transparent zebrafish (Fig 6A) confirmed the findings in IHC. The bacterin was detected in scale pockets and/or skin from 1 min pb to 12 hpb (Fig 6B–6D and 6F–6H) but not at 24 hpb. No reaction was detected in the gills at any time point. When the inner organs were excised from the fish only intestine and oesophagus showed presence of bacterin (Fig 6E). Control fish intestines showed some auto-fluorescence, however the auto fluorescence was less intense than the fluorescence emitted by the GFP-tagged Yr. To confirm the presence of the bacterin in the intestines of the bath vaccinated fish, the intestines were cut open and the intestinal fluids confirmed positive for the presence of GFP-Yr by visualisation using GFP filter settings.

#### Juveniles and larvae

Live imaging did not reveal any bacterin on the outside of either juveniles or larvae. The inner organs were not examined using this technique due to the small size of the fish.

## Discussion

Aquacultured salmonids are routinely immersion vaccinated against the devastating bacterial pathogen *Y*. *ruckeri*. The immersion induces a protective response but the route of antigen processing is still relatively unknown. In order to get a better understanding of uptake and antigen transport, a transparent zebrafish was used as a model organism for rainbow trout. Antigen uptake was investigated in these fish at different life stages following a bath vaccination with a bacterin based on formalin inactivated GFP-tagged Yr. Skin, scale pockets and the intestine were found to take up and process the bacterin and different locations of uptake, related to the developmental stage of the fish, were observed.

In the immature larvae innate immune factors are functional from one dpf [18,34]. Mature neutrophils and primitive macrophages are present from 24–30 hpf [35], which are the primary actors of the larval innate immune system [18]. Uptake of the bacterin was however only observed 24 hpb in the specialized enterocytes of the mid-intestine (Fig 4A and 4B) [36]. These specialised enterocytes are believed to be antigen presenting and to be analogous to M-cells of the mammalian intestine and they contain supranuclear vacuoles in which pinocytosed luminal contents can be stored [37]. It is known that the ability of neutrophils and macrophages to phagocytose bacteria differs among the bacterial pathogens [38] and in this study only an indication of gill uptake of the Yr bacterin was found (Fig 5A and 5B), even though the GFP-tagged bacterin surrounded the gills. Bacterin was also present on skin surfaces but was not taken up (Fig 5C), indicating that the inactivated bacteria may have been stuck in the mucosal layer on these surfaces. In the 20 dpf larvae the spleen contains large amounts of erythroblasts and is an immunologically immature organ not capable of capturing antigen [39], which may explain why the spleen did not stain positive for the bacterin at any sampling points. Chettri *et al*. (2012) found that rainbow trout yolk sac larvae did not take up Yr; only when the adaptive immune system became functional (fry) the fish became susceptible [40]. Results from this study indicate that zebrafish larvae may have similar immune regulations as rainbow trout and only when the fish becomes immunocompetent the Yr bacterin is taken up at other locations than the intestine.

Juveniles were sampled 54 dpf, where the immune system is more developed and almost fully mature. The zebrafish immune system has been estimated to be functionally and morphologically mature at 4–6 weeks post fertilisation (wpf) [41,42]. Just before the maturation of the immune system, metamorphosis takes place (2–4 wpf). In this stage larval fins are absorbed, the gut tube drops more ventrally and most important for this study the development of scales and pigmentation patterns begins [7,43,44]. The juvenile scale pockets adjacent to fin bases did not stain positive for the bacterin, which was the case for adults. This may indicate that the scales are under development and that the bacterin is not able to enter through the epidermal layer.

The nares stained positive for the bacterin at the bases of the sensory hairs (Fig 3B) at 2 hpb, which was never observed in the adult fish. This may indicate that throughout the development of the fish different immune tissues play more or less transient roles according to immune maturity. The nares have been found to include M-like cells and to be of importance for the rainbow trout immune responses [45]. The nose of zebrafish is an extra-thymic site and it may also be an antigen-presenting site but its immunological role before and after full maturation is reached at three months of age should be further investigated.

At 12 hpb the bacterin was visible in the spleen of the juveniles (Fig 3C), however there is less staining compared to the adult spleen two hpb. This may indicate that the spleen is under development and is capable of capturing small amounts of antigen.

In adults, the bacterin was evident in scale pockets, predominantly at fin bases (Fig 1A–1E and Fig 6C and 6D, 6F–6H). These inactivated bacteria are not actively adhering to the tissues, which indicate a passive entry into the host. In rainbow trout it has been shown that the bases of fins are major portal entry points of pathogens [46] and the same trend has been described in zebrafish [47,48]. Also from this study it appears as pathogens, dead or alive, enter passively or actively via scale pockets at the fin bases. We speculate that these scale pockets may be less concealed by the epidermis compared to scale pockets on other parts of the body, which may be due to fin movements increasing the risk of damaging the epidermal layer covering the scales.

The gills of adult zebrafish in this study were found to take up only small amounts of inactivated bacteria (Fig 2A). Other studies have shown that gills of zebrafish are a portal entry of pathogens [49] and that they take up nanoparticles and nanoliposomes [50–52]. The lack of visible uptake points towards specificity of antigen trapping in the gills, which is supported by a previous study, in which Yr bacterin and Yr O-antigen-labeled fluorescent beads were taken up by the gills, but unlabeled fluorescent beads were not [53]. On the other hand inactivation of the bacteria might be the cause of the low amount of uptake and other mechanisms might be involved, which warrants further studies.

The gut was the only place, where the uptake of bacterin was evident throughout the different life stages (Figs 1G–1I, 3A, 4A and 4B). These findings support the idea, that the intestine indeed is a major point of antigen entry as previously postulated [4,5,54].

Yr antigens were present in the spleen of the adult zebrafish from two hpb and onwards (Fig 1M–1O), indicating that the spleen is able to capture antigens. The spleen is a major lymphoid organ in teleosts containing lymphocytes, antigen presenting cells and antibody producing cells [35,55]. Lymphoblasts are evident in the spleen of zebrafish and developing ellipsoids are involved in the capture of antigen from three months of age [39].

The presence of antigen in the liver 24 hpb (Fig 1Z) may indicate that antigens, trapped in the gut, were transported via blood to the liver. Ohtani *et al*. (2015) suggested that this was the case in rainbow trout following immersion vaccination [5]. The presence of antigen detected in the kidney at 24 hpb (Fig 1T) is notable as the kidney is a major lymphoid organ [35]. The antigen may have been transported to the kidney from the spleen [56].

In this study zebrafish was used as a model organism for rainbow trout. The uptake at different life stages of zebrafish of an inactivated bacterium was investigated. Several of the uptake mechanisms appeared to be similar to rainbow trout uptake mechanisms even though there are deviations. Common for both species is the major uptake in the gut after swallowing the antigen [4,5,57]. Antigens enter through skin in rainbow trout [4,5,57] and the same trend is observed in adult zebrafish. The gills of 0.32 g rainbow trout fry were found to be a major uptake site of a Yr bacterin [5], whereas the gills of 4–5 g juvenile rainbow trout only took up small amounts compared to the lateral line and the gut [4]. Other studies have found the gills of rainbow trout to be a major uptake site [5,58]. Tatner *et al*. (1984) found that live *A*. *salmonicida* entered through gills of 1 g rainbow trout, whereas inactivated bacteria only entered to a limited extent [57]. In this study the zebrafish gill was not found to be a major uptake site of inactivated Yr in any of the life stages investigated even though gills of zebrafish have been shown to be major uptake sites [50]. In this respect, the antigen uptake mechanisms of zebrafish larvae differed from rainbow trout fry (0.32 g), whereas the later stages of both species appear to have more similar uptake means.

To increase the strength of this study a greater sample size per time point would be relevant together with a setup including rainbow trout of different ages, however that was not possible within this experimental operation.

Live imaging of adult zebrafish represents a non-invasive, gentle and easy way of investigating antigen uptake on the outside and in the skin of transparent zebrafish. This method is not suitable to investigate internal organs *in vivo* and is overall less sensitive that IHC.

In conclusion, it is apparent that different life stages of zebrafish have different immunological uptake strategies for Yr antigens. The innate immune system is the only one functioning in zebrafish larvae, and uptake is only seen in the intestine. The mature immune system is only fully developed at three months of age, illustrated by the presence of antigen in the spleen, liver and kidney, observed in this study. The basic functions of antigen processing of the zebrafish seem similar to rainbow trout and zebrafish may represent a suitable model organism for this kind of immunological studies for rainbow trout.

## Supporting Information

S1 Fig Section of a zebrafish showing organs and tissues relevant for the present study. G is gills, I is intestine, K is kidney, L is liver, N is nose, S is spleen, SP is scale pocket or skin.
(TIFF)

S2 Fig Immunohistochemical staining of adult zebrafish tissues following a sham bath. Sections were stained with an anti-*Y*. *ruckeri* antibody and the absence of colour reactions are shown for the scale pocket, the kidney, the spleen, the intestine, the liver and the gill. The scale bars are 100 μm long.
(TIFF)
