# Supplementary figures and images for: Antigen Uptake during Different Life Stages of Zebrafish (Danio rerio) Using a GFP-Tagged Yersinia ruckeri
Source: PLoS One. 2016 Jul 12;11(7):e0158968. doi: 10.1371/journal.pone.0158968 (PMC4942034; doi:10.1371/journal.pone.0158968)

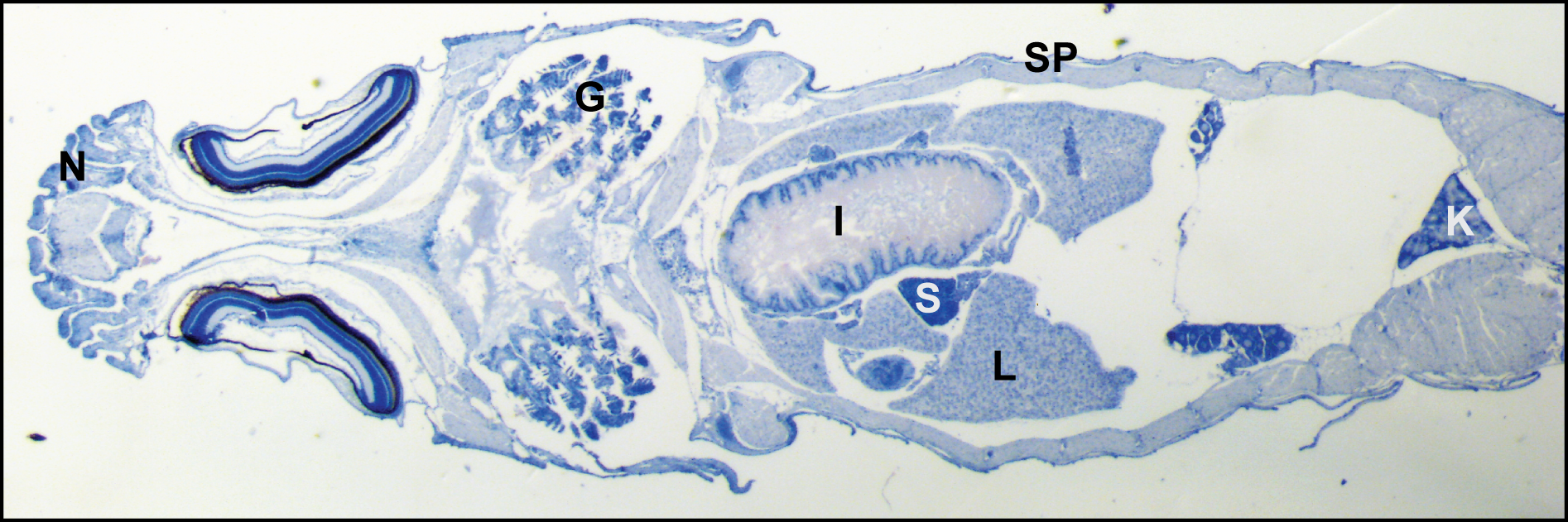

Supplement: S1 Fig — G is gills, I is intestine, K is kidney, L is liver, N is nose, S is spleen, SP is scale pocket or skin. (TIFF) [file pone.0158968.s001.tiff]

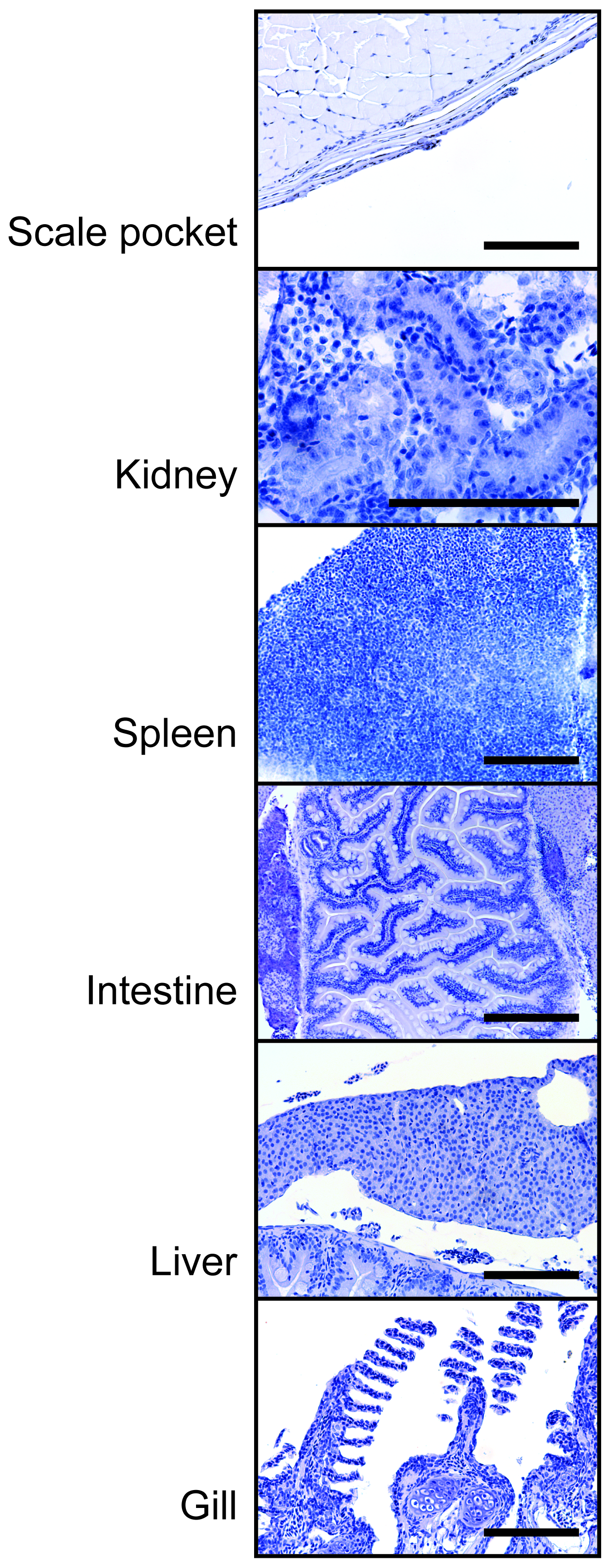

Supplement: S2 Fig — Sections were stained with an anti-Y. ruckeri antibody and the absence of colour reactions are shown for the scale pocket, the kidney, the spleen, the intestine, the liver and the gill. The scale bars are 100 μm long. (TIFF) [file pone.0158968.s002.tiff]
